# Reducing Overtreatment in Gynecologic Oncology: The Case for Less in Endometrial and Ovarian Cancer

**DOI:** 10.3389/fonc.2016.00118

**Published:** 2016-05-09

**Authors:** Sarah M. Temkin, Edward J. Tanner, Summer B. Dewdney, Lori M. Minasian

**Affiliations:** ^1^The Division of Cancer Prevention, The National Cancer Institute, National Institutes of Health, Bethesda, MD, USA; ^2^The Kelly Gynecologic Oncology Service, Johns Hopkins School of Medicine, Baltimore, MD, USA; ^3^Division of Gynecologic Oncology, Rush University School of Medicine, Chicago, IL, USA

**Keywords:** gynecologic cancer, ovarian cancer, endometrial cancer, overtreatment, cancer care delivery

## Abstract

A growing awareness of the harms of overtreatment in cancer care has reached physicians, patients, health policy makers, and medical researchers. Overtreatment exposes patients to the risk of adverse events from procedures or medications that were not necessary. This review examines common practices in gynecologic malignancies that are unlikely to produce direct benefit to patients with these malignancies, but are likely to produce harms. Specifically, we will explore the utility of lymphadenectomy and adjuvant radiation for women with early-stage endometrial cancer; and screening for recurrence and continuous chemotherapy for advanced-stage ovarian cancer patients.

## Introduction

The development of practical quality care measures has become a priority within the US health-care system. The Institute of Medicine (IOM) has identified three categories of health-care quality problems: underuse, overuse, and misuse (1–4). Underuse of effective therapies and misuse of services have received the most attention from quality improvement initiatives and patient safety efforts. However, recently, growing attention to an epidemic of overtreatment has been critically evaluated as problematic.

The term “overuse” in health care was introduced in 1991 in an editorial by Mark Chassin, as “the provision of health services when the risks outweigh their benefits” (5). The IOM first defined overuse in 1998 as the use of health-care resources and procedures in the absence of evidence that the service is beneficial (2). Overuse has been cited as a driver of the high cost of cancer in the United States (3, 4).

Overtreatment is a component of overuse defined as “treatment of conditions that will never cause symptoms, is futile or is excessive in complexity, duration, or cost when compared to accepted standards of care” (6). Overtreatment may seem innocuous in some cases (e.g., a patient has an extra blood test). In other cases (e.g., patients receiving chemotherapy or surgery that will not work for them), the harms are more apparent. Beyond the issue of cost, overtreatment exposes patients to the risks of adverse events from medications or procedures that they do not need.

In 2012, the American Board of Internal Medicine (ABIM), along with nine other specialty societies, released its Choosing Wisely campaign, focused on reducing overuse of specific medical tests or procedures in different health-care specialties. It includes an explicit goal of avoiding care that is “unnecessary or whose harm may outweigh the benefits” (7). The recommendations provided by the Society for Gynecologic Oncology (SGO) are listed in Table 1 (8). These SGO recommendations focus primarily on screening. Reducing overscreening and overtesting represents an important first step in controlling overuse. But these measures address low-hanging fruit – the most obvious or least remunerative examples of overused procedures.

**Table 1:** **The society for gynecologic oncology choosing wisely (8)**.

**•**	**Do not screen low-risk women with CA-125 or ultrasound for ovarian cancer** ○ CA-125 and ultrasound in low-risk, asymptomatic women have not led to diagnosis of ovarian cancer in earlier stages of disease or reduced ovarian cancer mortality. False-positive results of either test can lead to unnecessary procedures, which have risks of complication
**•**	**Do not perform colposcopy in patients treated for cervical cancer with Pap tests of low-grade squamous intraepithelial lesion (LGSIL) or less** ○ Colposcopy for low-grade abnormalities in this group does not detect recurrence unless there is a visible lesion and is not cost-effective
**•**	**Do not perform Pap tests for surveillance of women with a history of endometrial cancer** ○ Pap testing of the top of the vagina in women treated for endometrial cancer does not improve detection of local recurrence. False-positive Pap smears in this group can lead to unnecessary procedures, such as colposcopy and biopsy
**•**	**Avoid routine imaging for cancer surveillance in women with gynecologic cancer, specifically ovarian, endometrial, cervical, vulvar, and vaginal cancer** ○ Imaging in the absence of symptoms or rising tumor markers has shown low yield in detecting recurrence or impacting overall survival
**•**	**Do not delay basic level palliative care for women with advanced or relapsed gynecologic cancer, and when appropriate, refer to specialty level palliative medicine** ○ There is now an evidence-based consensus among physicians who care for cancer patients that palliative care improves symptom burden and quality of life. Palliative care empowers patients and physicians to work together to set appropriate goals for care and outcomes. Palliative care can and should be delivered in parallel with cancer directed therapies in appropriate patients

As cancer treatment becomes increasingly expensive with the rapid development of precision medicine, eliminating overuse is crucial to a sustainable system of care. Easily identifiable targets of overuse in gynecologic cancers beyond those identified in the Choosing Wisely campaign exist. The purpose of this review is to explore strategies to reduce overtreatment in gynecologic cancers. This review will focus on the following specific examples selected by the authors as high priority areas where overtreatment can be curtailed: (1) the practice of lymphadenectomy and (2) postoperative radiation in early-stage endometrial cancer; (3) screening for recurrence in asymptomatic ovarian cancer patients; and (4) continuous chemotherapy for the treatment of ovarian cancer. Recommendations for reducing overtreatment are provided by the authors as opinions.

## Endometrial Cancer

Endometrial cancer is expected to be diagnosed in 60,050 women in the United States in 2016, making it the most common gynecologic malignancy (9). Fortunately, most patients (80%) are diagnosed with local disease and will survive their cancer diagnosis. In fact, the majority of 5-year deaths in this population are due to the medical comorbidities frequently linked with endometrial cancer, including older age, obesity, and diabetes (10). Initial treatment includes a hysterectomy with salpingo-oophorectomy with surgical staging. Patients who develop metastatic recurrent disease have a poor prognosis and treatment options are limited (11). Because of the difficulty predicting who is at risk for recurrence, adjuvant treatment following hysterectomy is broadly prescribed to many with the goal of preventing recurrence in a few patients.

Although as a whole, clinical stage I endometrial cancer patients have a low risk for lymphatic metastasis, GOG 33 showed that some risk factors (grade, depth of myometrial invasion, and vascular space invasion) do correlate strongly with lymphatic metastasis (12). The primary role of surgical staging for endometrial cancer is to identify the small subset of patients with lymphatic metastasis to the pelvic lymph nodes as these are patients known to benefit from adjuvant therapy. Unfortunately, none of this information is reliably available pre- or even intra-operatively. The accuracy of preoperative tumor grade is poor, as 20–25% of cases that are grade 1 on biopsy will be upgraded after hysterectomy. Accuracy of depth of invasion at frozen section is similarly disappointing, especially when performed outside of high volume centers with experienced pathologists. A prospective, blinded study of the accuracy of frozen section revealed that tumor grade at frozen section correlated with final pathology in only 58% of cases, while depth of invasion correlated in 67% of patients. Overall, 28% of patients were upstaged from the intraoperative assessment to final pathology (13).

Surgical staging had been the norm for decades, its practice reinforced by retrospective studies showing a survival benefit in patients with high-risk features who underwent a systematic pelvic and para-aortic lymphadenectomy (14, 15). This surgical dogma has been questioned after two prospective randomized trials, evaluating that the value of lymph node dissection at the time of hysterectomy (ASTEC, CONSORT) failed to demonstrate a survival advantage with lymphadenectomy while confirming significant morbidity with the procedure (16, 17). The morbidity associated with lymph node dissection includes direct surgical morbidity (increased intraoperative time, greater blood loss, and risk of surgical complications) as well as the long-term consequences of lymphedema. Despite criticisms of these studies, including (1) a low-risk patient population with a low probability of nodal metastases and (2) the inconsistent application of information obtained from the nodal dissection to guide adjuvant therapy, these studies reflect many of the real world issues faced by clinicians caring for endometrial cancer patients.

Prospective studies examining the value of postoperative adjuvant treatment in patients with apparent early-stage disease but no lymphatic assessment have failed to demonstrate a survival benefit (11, 18–20). In the 1970s, a randomized prospective trial of postoperative whole pelvic radiation versus brachytherapy alone concluded that although radiation decreased recurrence rates, it had no effect on overall survival. More than 20 years of follow-up to this study revealed decreased long-term survival and increased secondary malignancies following postoperative whole pelvic radiation (21). The finding that the reduction of locoregional recurrence after pelvic radiation was not associated with a survival advantage has been confirmed subsequently by PORTEC-1, GOG-99, and ASTEC (11, 17, 19). The PORTEC-2 trial showed that vaginal brachytherapy provides equivalent locoregional control when compared to whole pelvic radiation with fewer adverse effects and improved quality of life (18). Low-risk patients (Stage 1A and B, Grades 1 and 2) randomized to vaginal brachytherapy or observation have been shown to have similar recurrence risks (22). Despite flaws in many of these trials (e.g., underpowered for a survival outcome), adjuvant radiation is not recommended for women with low-risk disease (23).

Early-stage endometrial cancer patients who are observed after surgery, although overall more likely to recur, are more likely to have isolated vaginal recurrences than women treated with adjuvant radiation (19–21, 24). Salvage rates for isolated pelvic recurrences with modern radiation techniques have been shown to be high (24, 25).

Trends in the use of lymphadenectomy in women who underwent surgery for endometrioid adenocarcinoma of the endometrium in the United States were recently analyzed using the surveillance, epidemiology, and end results (SEER) cancer registry between 1998 and 2012. Investigators found that a decreased frequency of lymphadenectomy from 2007 to 2012 was not associated with a change in the proportion of women found to have lymphatic metastasis. Interestingly, a small increase in survival accompanied the decrease in lymphadenectomy (26).

### The Identification of Endometrial Cancer Patients Who Can Safely Avoid Lymphadenectomy

Accurate identification of the small number of patients with lymphatic metastasis remains crucial as lymphadenectomy for these patients can have prognostic and possibly therapeutic value. When high-risk features are identified after hysterectomy only, clinicians must decide between returning to the operating room for a staging lymphadenectomy or basing treatment decisions on uterine factors, an approach that has a significant risk of resulting in overtreatment with postoperative adjuvant radiation as the majority of patients at “high risk” for lymphatic metastasis actually do not have metastatic disease (27).

Using a composite index of traditional risk factors for recurrence, investigators from the Mayo Clinic have identified a subgroup of patients at very low risk for lymph node metastasis. In a series of 328 patients treated at the Mayo Clinic with grade 1 or 2 endometrioid tumors, <2 cm in diameter and <50% myometrial invasion identified at time of intraoperative frozen section, the rate of nodal metastasis was 5% and 5-year survival was 97% (28). These criteria were further examined prospectively through a multi-institutional evaluation that noted a negative predictive value of 98.2% (29, 30). Broader utilization of these criteria could potentially eliminate unnecessary lymphadenectomies in women with endometrial cancer.

Sentinel lymph node biopsies (SLN) have been proposed as a surgical alternative to complete lymphadenectomy in patients with apparent early-stage endometrial cancer. The goal of SLN mapping is to accurately identify lymph node metastases while saving patients from the morbidity of complete lymphadenectomy. The utility of this technique as a strategy to reduce overtreatment has been firmly established in other disease sites (breast cancer and melanoma); however, the value and positive predictive value of SLN mapping in endometrial cancer has not been explored outside of single institution studies. Whether SLN biopsy can prevent the perioperative morbidity and long-term sequelae of lymphedema in this population without increasing recurrence rates and disease-related mortality is an important clinical question.

### The Identification of Endometrial Cancer Patients Who Can Safely Avoid Adjuvant Radiation

The strongest argument for lymphadenectomy in early endometrial cancer is that women with a lymph node sampling may be able to safely avoid adjuvant radiation. Information obtained from lymph node dissection influences the prescription of postoperative adjuvant radiation. A report of 181 women, with a diagnosis of grade 1 endometrial cancer, who underwent staging lymphadenectomy found that 19% of the neoplasms were upgraded, 18% were upstaged, while adjuvant therapy was affected by the results of lymphadenectomy in 26% of women (31). Women without surgical staging received radiation at higher rates than those with information available regarding their lymph node status. The application of newer surgical algorithms (using Mayo criteria to select patients who need lymphadenectomy) and validation of the practice of SLN could potentially reduce overprescribing of adjuvant radiation without resorting to lymphadenectomies for all.

Recent advances in molecular technology may provide additional insight into the identification of patients with a good prognosis who may not require treatment beyond a hysterectomy. The Cancer Genome Atlas (TCGA) performed an integrated molecular analysis of 373 endometrial tumors and was able to define four groups based on genomic characterization of mutation profiles, and to furthermore demonstrate that these groups correlate with prognosis. One of these groups (POLE-mutant) carried an exceptionally good prognosis (32). Between 4 and 12% of endometrial cancers are POLE-mutated and this genetic abnormality does not necessarily map onto traditional histopathological categories (32–35). Updated molecular classifications of disease to predict biologic behavior are needed. An assay able to identify patients at low risk for recurrence would transform treatment for this disease.

## Ovarian Cancer

Ovarian cancer is the most lethal of the gynecologic cancers. In the United States in 2016, there will be an estimated 22,280 cases and 14,240 deaths related to this disease (9). Among women with stage III or IV ovarian cancer, 5-year survival is only 10–25% despite aggressive treatment with surgery and adjuvant chemotherapy. Despite high response rates to initial therapy, epithelial ovarian cancer ultimately recurs in most patients. The poor prognosis associated with a diagnosis of epithelial ovarian cancer is due both to the advanced stage of disease at the time of diagnosis and the eventual development of chemotherapy-resistant disease. However, this disease is heterogeneous and there is considerable variability in survival even among patients diagnosed at an advanced stage (36). A reliable molecular signature identifying patients with a better or worse prognosis has not yet been identified.

Because of the high relapse rates, the follow-up of women treated for ovarian cancer represents a challenge for the gynecologic oncologist. Cancer surveillance following initial treatment and complete response is essentially screening for early relapse. In order for a screening test to be effective, effective treatment for the disease (in this case early relapse) must be available and the treatment of early relapse would have to be more effective than the treatment of late relapse. In the setting of platinum resistant disease, few therapeutic options prolong survival. In the setting of platinum-sensitive ovarian cancer, retreatment with platinum-based combination chemotherapy results in high complete response rates. As the platinum-free interval extends, the response rates to retreatment become even higher.

**Figure 1:**
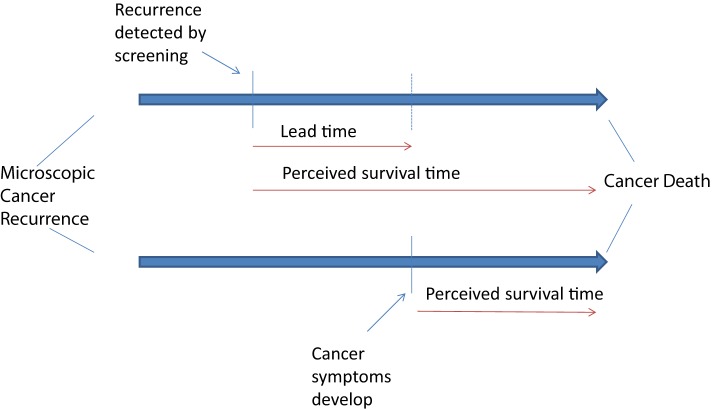
**Lead time bias that develops from early screening for cancer recurrence**.

One of the harms of this screening for recurrent ovarian cancer is the development of a lead time bias (Figure 1). Early diagnosis of recurrent disease exposes the patient to additional chemotherapy without this improving overall survival and may limit treatment further into the disease course. Many women with ovarian cancer receive multiple imaging tests per year and lifelong chemotherapy once recurrent disease is diagnosed. Both imaging and continuous chemotherapy are of little value in changing survival from this disease but are commonly prescribed (37, 38).

### The Utility of Following CA-125 Levels in Improving Survival from Ovarian Cancer

Cancer antigen 125 (CA-125) is a glycoprotein that is the current standard of care biomarker for ovarian cancer surveillance. More than 80% of women with advanced-stage ovarian cancer have an elevated level of CA-125 at the time of initial diagnosis. Periodic follow-up measurements of CA-125 levels after treatment for ovarian cancer allow the detection of recurrences months before symptoms or signs appear, since the CA-125 level generally increases 2–6 months before a recurrence is radiologically or clinically detectable (39). However, treatment for recurrence in asymptomatic women with an increased CA-125 level and a history of ovarian cancer has not been shown to alter survival. In a landmark study conducted by the European Organisation for Research and Treatment of Cancer (MRC OV05/EORTCC 55955), 529 women who experienced a complete clinical remission after undergoing initial treatment for ovarian cancer were randomized to undergo treatment for recurrence either (1) immediately after detection of a rise in the level of CA-125 or (2) after the onset of symptoms, regardless of the CA-125 level. No evidence of a survival benefit was found with early treatment for recurrence (27.1 versus 25.7 months) on the basis of an elevated CA-125 concentration. Women who were in the early group had earlier deterioration in quality of life versus women in the delayed treatment group (40). Criticisms of this study included enrollment of poor prognosis women, changes in second-line chemotherapy options for ovarian cancer, and low rates of secondary cytoreduction (41). A retrospective study of 121 patients undergoing secondary cytoreduction did suggest an advantage for patients whose recurrence was discovered through screening (42). This single institution study may have been biased by its retrospective nature and the tertiary care setting. Overall, there is no clear evidence that surveillance impacts survival compared to waiting for the presentation of symptoms (41, 43).

### The Utility of Routine Imaging in Improving Survival from Ovarian Cancer

Expert recommendations include obtaining imaging in patients with a history of ovarian cancer only in women with an increasing tumor marker or with symptoms worrisome for recurrence (41, 44). Computerized tomographic scans (CT) are currently the imaging modality of choice for the evaluation of suspected ovarian cancer recurrences. Given the lack of evidence that surveillance using the level of CA-125 present in blood samples prolongs survival, it is implausible that the early detection of recurrence through routine CT scanning could result in any significant increase in survival (40). These clinical limitations of CT make magnetic resonance imaging (MRI) and positron emission tomography (PET) imaging with their added expense and potentially increased false-positive rates challenging to justify in the asymptomatic ovarian cancer patient.

### Chemotherapy and the Treatment of Asymptomatic Disease

The majority of ovarian cancer patients will relapse within 5 years and, therefore, require salvage chemotherapy. Although improvements in chemotherapy have increased overall survival, recurrent ovarian cancer remains a lethal disease. Because salvage treatments are not curative, the goals of treatment in this setting are to extend survival through disease control and palliation of cancer symptoms. An emphasis on an individual patient’s quality of life is crucial.

A 2006 study evaluated ovarian cancer survival with the SEER database for patients treated with chemotherapy by a medical oncologist or gynecologic oncologist. Although both groups of physicians are trained to provide medical treatment to ovarian cancer patients, substantial differences in the patterns of care emerged based on the patient’s provider. During the first 5 years of care for ovarian cancer, patients treated by medical oncologists received more weeks of chemotherapy than patients treated by gynecologic oncologists (patient mean, 16.5 versus 12.1 weeks, respectively, *P* < 0.0023). This increase in chemotherapy administration translated to increased adverse events. Gynecologic oncology patients had fewer weeks that included chemotherapy-associated adverse events than medical oncology patients (patient mean, 8.9 versus 16.2 weeks, respectively, *P* < 0.0001). No survival advantage was achieved for patients receiving chemotherapy administered by a medical oncologist (37).

## Conclusion

As we strive toward defining quality measures in health care in the United States, defining best practices for women with gynecologic cancer should be a priority. Further research in endometrial cancer should be focused on defining which women with this disease can safely avoid lymphadenectomy and post-hysterectomy radiation and providing evidence that postoperative surveillance is safe such that practitioners feel comfortable making this recommendation. Best practices for post-treatment surveillance in ovarian cancer patients should be individualized, taking into account the clinical benefit of second-line therapy, costs, morbidity and mortality of the surveillance methods, available treatment options, and patient preference.

## Author Contributions

All authors listed, have made substantial, direct and intellectual contribution to the work, and approved it for publication.

## Conflict of Interest Statement

The authors declare that the research was conducted in the absence of any commercial or financial relationships that could be construed as a potential conflict of interest.
